# Relationship Between Somatic Cell Counts and Mammary Gland Parenchyma Ultrasonography in Buffaloes

**DOI:** 10.3389/fvets.2022.842105

**Published:** 2022-03-21

**Authors:** Xinxin Zhang, Muhammad Jamil Ahmad, Zhigao An, Kaifeng Niu, Wei Wang, Pei Nie, Shan Gao, Liguo Yang

**Affiliations:** ^1^Key Laboratory of Animal Genetics, Breeding and Reproduction, Ministry of Education, College of Animal Science and Technology, Huazhong Agricultural University, Wuhan, China; ^2^International Joint Research Centre for Animal Genetics, Breeding and Reproduction (IJRCAGBR), Huazhong Agricultural University, Wuhan, China; ^3^Hubei Province's Engineering Research Center in Buffalo Breeding and Products, Wuhan, China

**Keywords:** SCC, NPVs, PSD, ultrasonography, mastitis

## Abstract

The aim of the present study was to determine whether the echotextural features of the mammary gland parenchyma in buffaloes during lactation at different somatic cell levels could be used to diagnose mastitis. This study was divided into two parts. In the first experiment, experimental buffaloes (*n* = 65) with somatic cell counts (SCC) tests (*n* = 94) in different seasons, including spring (*n* = 22), summer (*n* = 24), autumn (*n* = 37), and winter (*n* = 11), were used to obtain ultrasonic variables for each quarter of mammary gland that could best explain the corresponding somatic cell level. In the second part of the study, the first part's experimental results were verified by subjecting at least one-quarter udder of eight buffaloes to ultrasonography seven times during mid-July to mid-August for obtaining ultrasonic values at different somatic cell levels. The echo textural characteristics [mean numerical pixel values (NPVs) and pixel heterogeneity (pixel standard deviation, PSD)] were evaluated using 16 ultrasonographic images of each buffalo with Image ProPlus software. The effects of SCC, days in milk (DIM), scanning order (SO), season, as well as the scanning plane and udder quarter (SP + UQ) on both the PSD and NPVs of the mammary gland were significant (*p* < 0.05). The correlation coefficient between pre-milking sagittal PSD and somatic cell score (SCS) was the highest (*r* = 0.4224, *p* < 0.0001) with fitted linear model: *y* = 0.19445x (dependent variable: SCS, independent variables: pre-milking sagittal PSD; *R*^2^ = 0.84, *p* < 0.0001). In addition, SCC and ultrasonic of udder quarter were followed for 1 month, confirming that pre-milking sagittal PSD of mammary gland value could explain the SCC variation in milk. The current study demonstrated that the ultrasonographic examination of the udder could be one of the complementary tools for diagnosing subclinical mastitis in buffaloes.

## Introduction

Buffalo is the second-largest milk-producing livestock, with an average of 118.162 million tons of buffalo milk per year over the last decade, according to the FAO statistical database (1). Like cows, mastitis is the most serious disease that causes economic loss in the buffaloes (2, 3). Although buffaloes are traditionally thought to be more resistant to mastitis than cows, some characteristics such as higher nutrient content in milk suitable for rapid microbial growth and pendulous udder with longer teats make buffaloes more prone to mastitis (2). In Nepal, the prevalence of subclinical mastitis is as high as 78% in buffalos and 55% in cows (4). Mastitis has a negative effect on milk composition (protein, fat, lactose, and mineral) (5, 6), curdling (7), milk yield (7), and conception rate (suppression to both corpus luteum (CL) diameter and function) (8) in buffaloes. Buffalos in developing countries are mostly raised on a small scale with adequate resources; limited quality research about health, management, nutrition, applied reproduction, and basic physiology (9) makes mastitis detection more difficult due to prevailing environmental and management conditions. Therefore, establishing the methods for early mastitis detection is significant for preventing and treating mastitis.

Early and accurate diagnosis of the disease is an essential part of treatment. Nowadays, many different methods for diagnosing mastitis are being practiced based on SCC and plate-culture techniques (10). SCC is an important indicator for judging subclinical udder infection; mastitis quarters included were defined as clinical mastitis or subclinical mastitis by SCC ≥ 200,000 cells/ml in the European Union, ≥250,000 cells/ml in Australia, and ≥150,000 cells/ml in New Zealand (11). Because of the significance of early diagnosis and early treatment, it is imperative to establish an economical, accurate, and rapid method for diagnosing mastitis on the farm (12).

Ultrasonography is a non-invasive technique that can examine udder for hematoma, abscess, inflammation, milk stones, tissue proliferation, congenital malformation, mucosal lesions, and foreign bodies (13). In recent years, ultrasound has been widely used to examine the anatomical structure of the mammary gland of various animals (14–16). Diagnostic ultrasonography has been employed to get the images of udder structures, including mammary glands with the mammary parenchyma, the lactiferous ducts, the mammary vessels, the teat (teat cistern and teat arteries), the supra-mammary lymph nodes, and the gland cistern (17). Several studies have investigated the relationship between the ultrasonography assessed mammary gland and SCC or mastitis (18–20). Ultrasound technology is considered an effective method for the rapid detection of mastitis.

Ultrasonography facilitates researchers in examining the same animals repeatedly and readily, providing grayscale and two-dimensional images of tissues in real time to evaluate their structure, echogenicity, and homogeneity. Therefore, the objective of this study was to develop a rapid method to detect mastitis by establishing the relationship between somatic cells and mammary echotextural characteristics in buffaloes.

## Materials and Methods

### Experimental Animals

The present investigation was carried out in the buffalo farm of Jinniu Animal Husbandry Co. Ltd., China, from November 2020 to September 2021. The experimental animals were divided into two groups: experimental group (EG, *n* = 65, test-day records = 94) was tested in the first part and the validation group (VG, *n* = 8, test-day records *n* = 54) with at least one-quarter udder was tested in the second part to verify the results of the first part. All lactating buffaloes were housed in the free-stall pens and fed a total mixed rations (TMR) diet. DIM of all the buffaloes enrolled in this study were between 5 and 349 days with the parity between 1 and 6 and milked twice a day, morning and evening, in a parallel milking parlor (6 × 2). The milking machine parameters were set to an operational vacuum of 50 kPa, 64 ± 3 pulsation cycles/min at a ratio of 60:40, which were maintained every 3 months. Data for individual buffalo data were obtained from herd management software (Daily management system for buffalo V1.0).

### Milk Sampling

All milk samples from the quarter mammary gland were collected on the morning of the ultrasound measurement, and the first few streams of milk were discarded before collection. Quarter-level SCC milk samples were collected in 50-ml sterile vials with preservatives added in advance that submitted to the Dairy Herd Improvement of Hubei testing center under −60°C conditions for SCC detection by somatic cytometer (CombiFoss FT+ FOSS Analytical, Hillerød, Denmark).

### Mammary Gland Measurements

**Figure 1:**
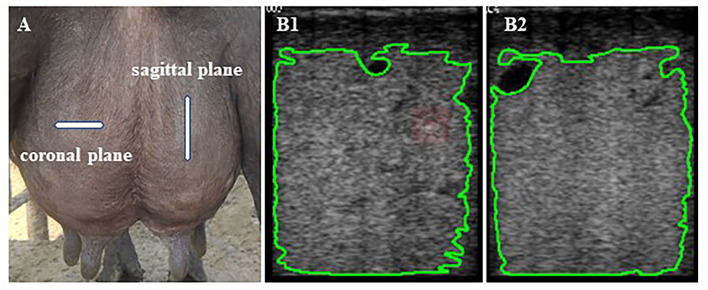
**(A)** The probe is located on the coronal and sagittal planes surfaces of the udder quarters. **(B1,2)** The mammary parenchyma echotexture of the selected area of coronal **(B1)** and sagittal planes **(B2)** excluding the areas with lactiferous ducts, gland cisterns, and visible blood vessels formations was calculated using Image-Pro Plus software.

It was challenging to interpret the sensitivity difference between 5- and 7.5-MHz probes in examining the mammary parenchyma (21). Previously, Wang et al. (22) recommended using linear array transducers with high frequencies in conventional ultrasound examinations for superficial organs such as the udder. A high-frequency probe produces a good-quality ultrasound image (23). Hence, real-time B-mode ultrasonography (WED-3000-v, equipped with LNA/6.5 MHz rectal probe, Shenzhen Well; D Medical Electronics Co., Ltd., Guangdong, China) working at 7.5-MHz linear array transducer was used at a depth of 90 mm to examine the udder area of all four mammary glands in lactation buffalo. The same experienced operator did all mammary ultrasound examinations. Mammary ultrasound was consistently examined in the following order: left front, left rear, right rear, and right front using a probe coated with ultrasound gel to enhance contact against the animal's skin. Udders were clipped of excess hair to reduce the influence of hair on ultrasound scan results. The probe was placed directly in the middle of the udders. Ultrasonographic scans of the coronal and sagittal planes of each of the four quarters of the udder were taken immediately before and after milking (Figure 1A).

Each buffalo was scanned 16 times, and a total of 1,710 ultrasound images were captured and saved in bitmap (BMP) format. Subsequently, images were transferred using universal serial bus (USB) for computer analysis with USB software Image-Pro Plus 6.0 (National Institutes of Health, Rockville Pike, MD, USA) to analyze, process, and edit grayscale images by pixel value statistics and calculate area to get the intensity values. Areas with lactiferous ducts, gland cisterns, and visible blood vessel formations were not considered for the grayscale analysis (Figure 1B). Grayscale ultrasound images are composed of many luminance elements (pixels) corresponding to multiple acoustic interfaces within the detected tissues (24). Numerical pixel values (NPVs) were defined as a quantitative measure of pixel brightness, and pixel heterogeneity [pixel standard deviation (PSD)] was the standard deviation of NPV in the region of interest. The mean NPVs and pixel heterogeneity (PSD) values were calculated based on the brightness (absolute black, 0, to absolute white, 255) of the selected area of each ultrasound image (25). The echotextural characteristics of mammary gland parenchyma (pre-milking coronal NPVs, pre-milking sagittal NPVs, pre-milking coronal PSD, pre-milking sagittal PSD, post-milking coronal NPVs, post-milking sagittal NPVs, post-milking coronal PSD, and post-milking sagittal PSD) were obtained by combining ultrasonic image and computer software.

### Ultrasonography of Udder Quarter at Different SCC Levels

To verify the echotextural variable's accuracy in detecting mastitis, at least one of the four udders of eight buffaloes of parity (2–5) with days in milk (DIM, 25–224), milk yield (MY mean, 6.01), and SCC tests (*n* = 54) was examined based on SCC tests (>200,000 cells/ml) of quarter milk samples. Mastitis can transfect to other quarter udder regions if not treated. Collecting milk samples and mammary glands ultrasound examination were carried out seven times at an interval of 6 and 1 day, respectively and followed up for 30 days to obtain mammary parenchyma echotexture images at different somatic cell levels.

### Statistical Analysis

#### Factors Affecting Mammary Gland Echotexture

The data from 94 test-day records from 65 buffaloes were included in calculating the fixed effects using SAS's general linear model (GLM) procedure implemented in SAS version 9.4 (SAS Institute Inc., Cary, NC, USA) (26). The main effects are DIM, parity, season, SCC, scanning plane for each quarter, and scanning order (SO). The echotextural variables were determined by GLM as follows:


$$\begin{array}{l} {\text{Y}}_{\text{ijklmn}}=\mu +{\text{DIM}}_{\text{i}}+{\text{Parity}}_{\text{j}}+{\text{Season}}_{\text{k}}+{\text{SCC}}_{\text{l}}+{\text{SO}}_{\text{m}}\\ +{\left(\text{UQ}+\text{SP}\right)}_{\text{n}}+{\left[\text{SO}\times \left(\text{UQ}+\text{SP}\right)\right]}_{\text{mn}}+{\text{e}}_{\text{ijklmn}} \end{array}$$

where Y_ijklmn_ is the NPVs or PSD; μ is the population mean of the model; DIM is fixed effect of lactation period (*i* = 1–4, the first class is DIM in 5–90 days and followed by classes of 90 days each, until the fourth class is 271–349 days); parity is fixed effect of the number of lactations (*j* = 1 to 3, primiparous were first levels, 2–3 parities were second levels, and 4–6 parities was the third level); season is fixed effect season of measurement [(*k* = Spring (April), Summer (July and August), Autumn (November), and Winter (January)]; SCC is fixed effect of somatic cell counts (*l* = 1–5, 1: <200,000 cells/ml, 2: 200,000–500,000 cells/ml, 3:500,000–800,000 cells/ml, 4:800,000–5,000,000 cells/ml, and 5:>5,000,000 cells/ml); SO is fixed effect of SO (*m* = 1 to 2, before or after milking); UQ + SP is fixed effect of scanning plane for each quarter (*n* = l to 8, sagittal vs. coronal within left front vs. left back vs. right back vs. right front udder quarter); SO × (UQ + SP) is the fixed effect of the interaction between SO and UQ + SP; and e_ijklmn_ is the random residual.

#### Correlation Analysis and Linear Regression Between SCS and Mammary Gland Echotexture

For normality and homogeneity of the SCC variable, the transformation formula SCS = 3 + log_2_ (SCC/100,000) was used, using only data from the 94 test-day records from 65 buffaloes. Pearson correlations analysis was performed using the PROC CORR in SAS (version 9.4, SAS Institute Inc., Cary, NC, USA) (27). DIM, parity, and season were added to the process as a covariate based on factors affecting mammary gland parenchyma echotexture. According to the correlation analysis results, the general linear regression model was fitted by PROC REG procedure to determine the association between SCS (dependent variable) and mammary gland parenchyma echotexture (independent variables).

#### Changes of Mammary Gland Echotexture at Different SCC Levels

Mean values of mammary gland echotexture at different somatic cell levels (<200,000 cells/ml, 200,000–5,000,000 cells/ml, and >5,000,000 cells/ml) were obtained after 1-month follow-up and compared by ANOVA using SAS (version 9.4, SAS Institute Inc., Cary, NC, USA) (28). The data were visualized using the software package GraphPad Prism 6 (version 6.0c; GraphPad Software, Inc., La Jolla, CA, USA) (29).

## Results

### Factors Affecting the Echo Textural Characteristics of Mammary Gland Parenchyma

Table 1 shows that the effects of SCC, DIM, SO, season, as well as the UQ + SP on both the PSD and NPVs of mammary glands were significant (PSD: SCC, *p* < 0.0001; DIM, *p* = 0.0101; SO, *p* = 0.0003; Season, *p* < 0.0001; UQ + SP, *p* < 0.0001; NPVs: SCC, *p* = 0.0478; DIM, *p* < 0.0001; SO, *p* = 0.0406; Season, *p* < 0.0001; UQ + SP, *p* < 0.0001). Whereas, parity and SO × (UQ + SP) on PSD were not found to have an effect on any of the measurements (parity, *p* = 0.4901; SO × (UQ + SP), *p* = 0.2901). Parity and SO × (UQ + SP) were found to have an effect on NPVs (parity, *p* < 0.0001 − SO × (UQ + SP), *p* = 0.0283).

**Table 1:** Factors affecting the echotextural characteristics of mammary gland parenchyma (NPVs and PSD).

**Effect**	**PSD**	**NPVs**
SCC (cells/ml)	<0.0001	0.0478
DIM/day	0.0101	<0.0001
Parity	0.4901	<0.0001
Season	<0.0001	<0.0001
UQ + SP	<0.0001	<0.0001
SO	0.0003	0.0406
SO × (UQ + SP)	0.2901	0.0283

*NPVs, numerical pixel values; PSD, pixel standard deviation or pixel heterogeneity; SCC, SCC categories; DIM, days in milk; UQ + SP, fixed effect of scanning plane for each quarter; SO, fixed effect of scanning order; SO × (UQ + SP) is the fixed effect of the interaction between SO and UQ + SP*.

**Figure 2:**
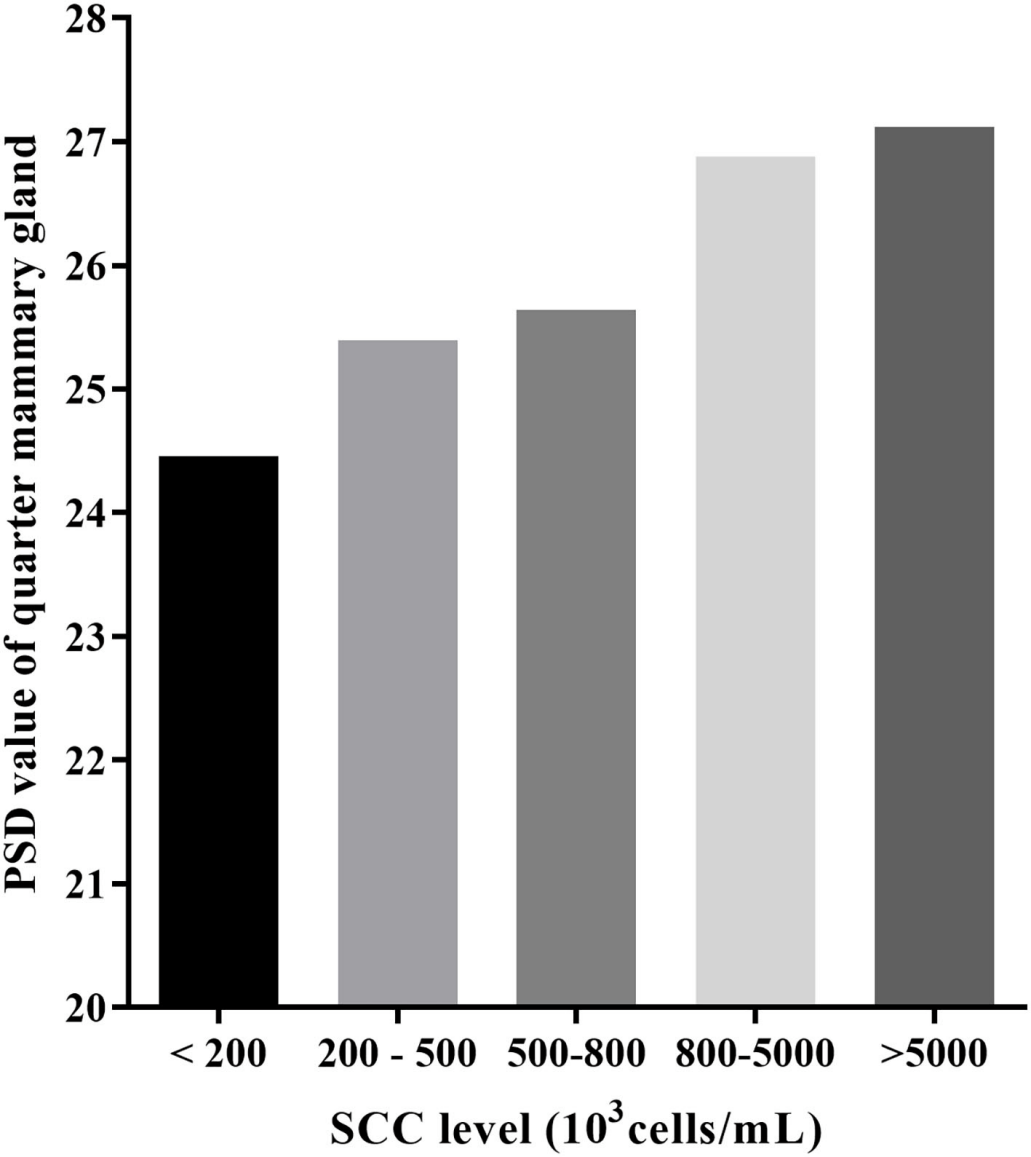
The least square means of PSD by SCC categories for buffaloes.

Mean PSD was the lowest in the SCC <200,000 cells/ml and increases with SCC increase (24.46 vs. 25.40 vs. 25.64 vs. 26.88 vs. 27.12; Table 2 and Figure 2). Mean PSD value of front half udder was larger than that of rear udder half, while mean NPV value was the opposite (PSD: 26.72 vs. 25.09; NPVs: 60.30 vs. 63.36; front half udder compared with rear udder half, respectively). During summer, PSD value was the highest compared with other seasons (summer: 27.17 vs. spring: 25.37 vs. autumn: 24.98 vs. winter: 26.07; *p* < 0.05). NPV value in spring was significantly lower than that in other seasons (spring: 47.74 vs. summer: 64.04 vs. autumn: 66.18 vs. winter: 69.35; *p* < 0.05). PSD were greater after than before milking, but NPVs were the opposite (PSD: 25.53 vs. 26.27, *p* < 0.05; NPVs: 62.73 vs. 60.93, *p* < 0.05; before compared with after milking, respectively). PSD and NPVs were highest between 5 and 90 days of lactation (PSD: 26.56; NPVs: 72.96).

**Table 2:** The least square means of NPVs and PSD by SCC, DIM, season, UQ + SP, and SO categories for buffaloes (Mean ± SEM).

**Effect**	**PSD**	**NPVs**
**SCC (cells/ml)**
<200,000	24.46 ± 0.24	61.38 ± 1.01
200,000–500,000	25.40 ± 0.31	59.81 ± 1.33
500,000–800,000	25.64 ± 0.44	61.51 ± 1.89
800,000–5,000,000	26.88 ± 0.23	63.91 ± 0.98
>5,000,000	27.12 ±0.37	62.54 ± 1.56
**DIM/day**
5–90	26.56 ± 0.18	72.96 ± 0.76
91–180	25.75 ± 0.24	64.63 ± 1.03
181–270	25.85 ± 0.40	55.25 ± 1.69
271–349	25.43 ± 0.44	54.47 ± 1.85
**Parity**
1	25.95 ± 0.24	57.05 ± 1.00
2–3	25.72 ± 0.21	63.57 ± 0.90
4–6	26.02 ± 0.33	64.87 ± 1.39
**Season**
Spring (April)	25.37 ± 0.24	47.74 ± 1.01
Summer (July and August)	27.17 ± 0.28	64.04 ± 1.21
Autumn (November)	24.98 ± 0.24	66.18 ± 1.00
Winter (January)	26.07 ± 0.41	69.35 ± 1.75
**UQ** **+** **SP**
Left front coronal	26.43 ± 0.34	60.11 ± 1.45
Left front sagittal	27.09 ± 0.34	60.38 ± 1.45
Left back coronal	24.57 ± 0.35	57.41 ± 1.48
Left back sagittal	24.68 ± 0.35	63.90 ± 1.50
Right back coronal	25.55 ± 0.33	63.73 ± 1.39
Right back sagittal	25.54 ± 0.33	68.38 ± 1.40
Right front coronal	25.83 ± 0.33	59.27 ±1.39
Right front sagittal	27.51 ± 0.33	61.45 ±1.39
**SO**
Before milking	25.53 ± 0.22	62.73 ± 0.95
After milking	26.27 ± 0.22	60.93 ± 0.93

### The Relationship Between Echotextural Characteristics of Mammary Gland Parenchyma and SCS

Four PSD traits were highly significant and positively correlated with SCC among echotextural characteristics of the mammary gland parenchyma and SCC of the milk obtained in the period before and after milking, respectively (*p* < 0.0001), but non-significant with NPVs (p > 0.05), respectively (Table 3). The correlation coefficient between pre-milking sagittal PSD and SCS was the highest (*r* = 0.4224, *p* < 0.0001), compared with pre-milking coronal PSD (*r* = 0.30734, *p* < 0.0001), post-milking coronal PSD (*r* = 0.27354, *p* < 0.0001), and post-milking sagittal PSD (*r* = 0.26595, *p* < 0.0001). The high value of adjusted *R*^2^ (0.82 to 0.84) indicated that the linear model of PSD fitting explained sufficient variability in SCC in milk. Pre-milking sagittal PSD had explained higher variation (*Y* = 0.19445 ×, adjusted *R*^2^ = 0.84) and fitted better than other models (Table 4).

**Table 3:** Pearson correlations coefficients between echotextural characteristics of mammary gland parenchyma and SCS (*n* = 94 test-day records).

**Variable**	***r***	***p*-value**
Pre-milking coronal NPVs	0.00626	0.9129
Pre-milking coronal PSD	0.30734	<0.0001
Pre-milking sagittal NPVs	−0.00828	0.885
Pre-milking sagittal PSD	0.4224	<0.0001
Post-milking coronal NPVs	0.00126	0.9825
Post-milking coronal PSD	0.27354	<0.0001
Post-milking sagittal NPVs	0.02105	0.7128
Post-milking sagittal PSD	0.26595	<0.0001

**Table 4:** The linear model between SCS and pre-milking sagittal, pre-milking coronal PSD, post-milking coronal PSD, and post-milking sagittal PSD.

**Input variable**	**Adjusted *R*^2^**	**Equation**	***p*-Value**
Pre-milking sagittal PSD	0.84	*Y =* 0.19445x	<0.0001
Pre-milking coronal PSD	0.82	*Y =* 0.20065x	<0.0001
Post-milking coronal PSD	0.82	*Y =* 0.19165x	<0.0001
Post-milking sagittal PSD	0.82	*Y =* 0.18708x	<0.0001

### Changes of Mammary Gland Echotexture of Quarter-Level at Different SCC Levels

**Figure 3:**
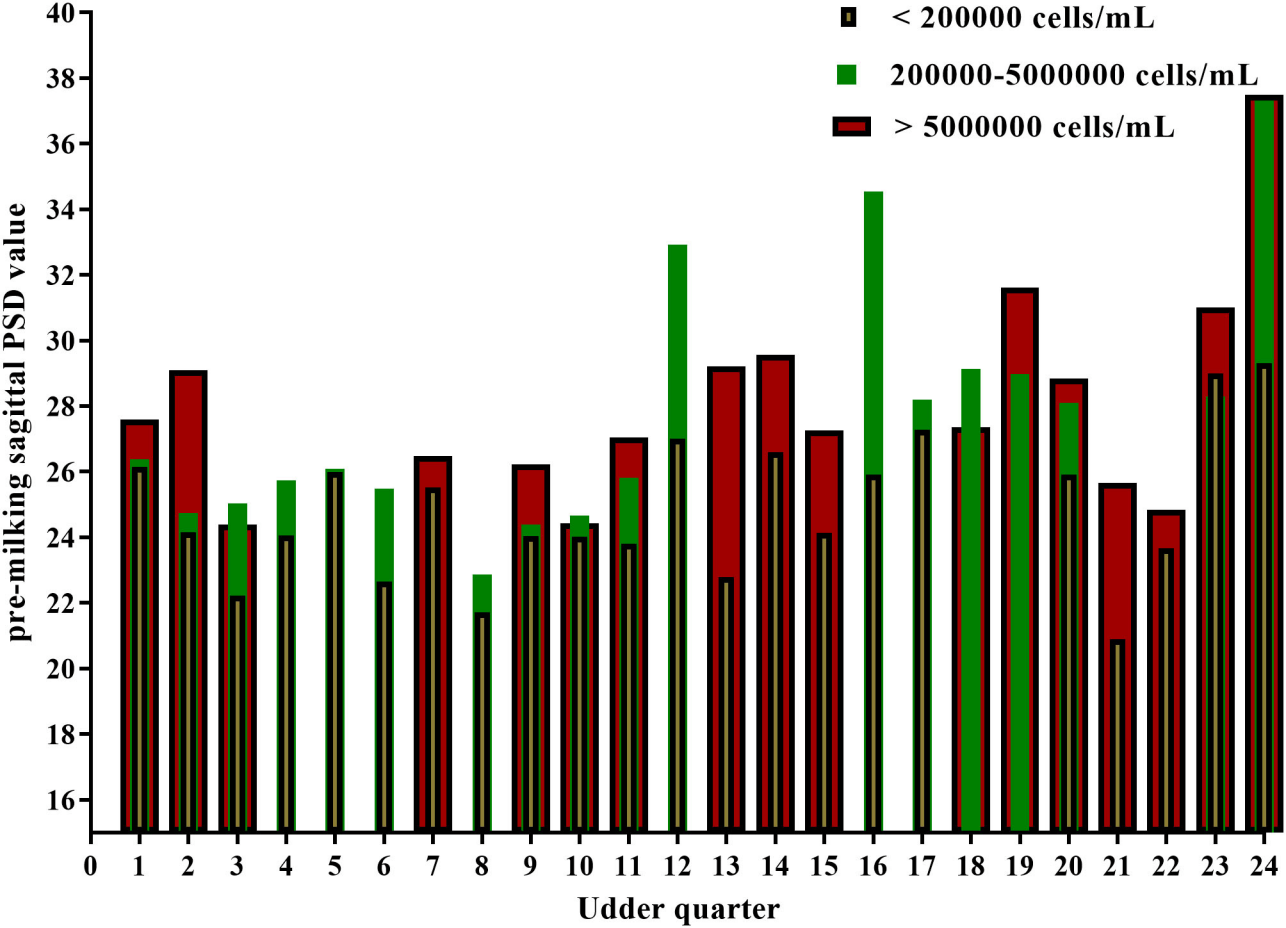
Pre-milking sagittal PSD values of 24 quarter-level udders at different somatic cell levels.

All the data were reported as the average of the same SCC level in seven ultrasonic testing and SCC levels varied in 24 out of 32 mammary glands. The PSD value for most quarter udder increases with somatic cells (Table 5 and Figure 3). A one-way ANOVA test was used to compare the pre-milking sagittal PSD values for different levels of SCC. The results showed that pre-milking sagittal PSD varied significantly (*p* = 0.003) between different levels of SCC. The pre-milking sagittal PSD value reached the lowest value at SCC <200,000 cells/ml and significantly lower than other levels (SCC <200,000: 24.85 vs. 200,000–5,000,000: 27.70 vs. SCC > 5,000,000: 28.12, *p* < 0.01).

**Table 5:** Descriptive statistics pre-milking sagittal PSD of the mammary gland in a single udder quarter using ANOVA analysis by SCC (54 test-day records for eight buffaloes).

**Quarter-level** **udders**	**SCC (10**^**3**^ **cells/ml)**			***p*-Value**
	**<200**	**200–5,000**	**>5,000**	
1	26.15	26.38	27.59	0.003
2	24.16	24.74	29.11	
3	22.23	25.03	24.38	
4	24.05	25.74	N	
5	25.98	26.09	N	
6	22.65	25.48	N	
7	25.51	N	26.48	
8	21.72	22.87	N	
9	24.04	24.38	26.22	
10	24.02	24.66	24.43	
11	23.81	25.81	27.04	
12	27.00	32.93	N	
13	22.79	N	29.22	
14	26.59	N	29.57	
15	24.13	N	27.25	
16	29.00	28.30	31.02	
17	25.90	34.54	N	
18	29.31	37.32	37.49	
19	27.28	28.21	N	
20	N	29.13	27.36	
21	N	28.99	31.61	
22	25.91	28.11	28.84	
23	20.89	N	25.65	
24	23.66	N	24.83	
Mean ± SEM	24.85 ± 0.47^b^	27.70 ± 0.90^a^	28.12 ± 0.78^a^	

ab *Pre-milking sagittal PSD means within the same row with different superscripts differ at P < 0.05*.

*N: There is no record of a match*.

## Discussion

The quality of feed, milking equipment, health conditions, and management systems of buffalo are all-inferior to those of cows, which significantly affects the prevention, diagnosis, and treatment of mastitis in buffalo, especially at the farm level. California mastitis test for diagnosing mastitis has the advantage of being quick and easy to perform on the farm. However, it results in subjective and indirect determination, which instigates finding more accurate techniques (30). The effectiveness of ultrasound for detecting mastitis in buffalo was first confirmed (14). Ultrasound has been used as a diagnostic tool for several decades. However, its use in buffalo just started in the last decade, and there is a shortage of literature on the relationship between ultrasound and mastitis in buffaloes (31). The purpose of this study was to determine the relationship between mammary gland echotexture values and SCC in milk and finally to find a simple and accurate diagnosis method for buffalo mastitis. To the author's knowledge, this was the first attempt to correlate quantitative echotextural characteristics of the udder with SCC in buffaloes.

Udder fibrosis and atrophy due to chronic mastitis had hyperechoic cordial bands representing the fibrous tissues, replacing the glandular tissues in buffaloes (14). The ultrasonographic examination may explain internal structure changes in the mammary gland and teat tissue of animals with fibrosis and mastitis (14). The characteristic changes of mammary glandular parenchyma, teat, and the milk that occurred during all different mastitis phases show the different intensities of echogenicity (32). The grayscale intensity values of mammary parenchyma in pregnancy toxemia were significantly greater than in healthy controls. Not only in the mammary gland, pixel intensity and pixel heterogeneity in the testicular parenchyma was also related to male reproductive health (33, 34).

Currently, SCC, either from the whole udder or quarter, has been the best indicator of the mammary glands' inflammatory status (30, 35). A previous study has reported that the image of the udder with mastitis appeared as hyperechoic parenchyma in buffaloes (36). The current study showed a weak correlation between SCS and NPVs, which may be caused by mastitis, leading to local hyperechoic mammary ultrasound without a significant increase in the mean value of NPVs. According to various studies, the ultrasonography of normal mammary parenchyma depicts a homogenous structure (17, 37, 38). On the contrary, mammary glands with mastitis reveal non-homogenous regions in mammary gland parenchyma from hypo- to hyperechoic (23, 39). Believing that lack of milk secretion makes visualization of the mammary structures more complex, they suggested performing an ultrasound at least 2 h after milking for optimal visualization. These support the present study results; the correlation coefficient between PSD value and SCS was more significant before milking. In addition, SCC and ultrasonic of udder quarter were followed for 1 month, confirming that pre-milking sagittal PSD of mammary gland value could explain the SCC variation in milk.

The present study found that examining the structures of the mammary gland parenchyma with vertical positioning showed better results than the horizontal direction, contradictory to findings in goats (39). However, Schwarz et al. (40) reported that examining the vertical plane of the mammary gland was more suitable and could describe the significant correlation between echotextural variables and milk composition (protein and lactose during both milking periods).

Our study showed that echotextural characteristics of the mammary parenchyma were influenced by many factors, including DIM, seasons, and measurement methods. Therefore, these factors should be considered while interpreting the ultrasonic-related effects. In our study, PSD values of the mammary gland were highest at 5–90 days of lactation and in summer. Coincidentally, Riekerink et al. (41) found that bulk milk SCC peaked in August to September during 4 consecutive years. Further, Morse et al. (42) found that clinical mastitis increases with increased temperature–humidity index value in summer. Hossein-Zadeh and Ardalan (43) reported that the odds of clinical mastitis increased in the first month of lactation. Early lactation mastitis occurs because of unresolved intramammary infections or the formation of intramammary infections during the dry period. A study in cows by Barkema et al. (44) shows that rear quarters were more likely than front quarters to have mastitis and high SCC. On the contrary, Hammer et al. (45) found that a quarter position is not associated with the risk of developing mastitis. However, the PSD value in the front quarters was higher than that in the rear quarters, which was confirmed in the study of Ali et al. (46), who found that the prevalence of mastitis was higher in the front quarters compared to the rear quarters in buffalo (prevalence of sub-clinical mastitis: front quarters 98/400 vs. rear quarters 85/400).

Interestingly, the results of our study showed that, before milking, the NPVs values were significantly higher than after milking, which was different from the results in cows (40). In agreement with current study findings, the PSD value of the mammary glands was larger after milking than before milking in Olkuska ewes (21). The variation in parenchymal echotexture can be explained by the differences in milk chemical composition and parenchymal tissue microstructure among different animals (21). The difference in the NPVs between dairy cows and buffaloes could be due to the much higher milk yield of cows than buffalo [34.71 kg/days vs. 9.01 kg/days (47, 48)]. A small amount of buffalo milk as fluids with a high total solid and fat content (49) plays the role of an acoustic window and without significantly reducing mammary grayscale intensity values.

## Conclusion

Despite the availability of different traditional and novel diagnostic tools for early mastitis diagnosis, there is no gold for testing as all tools have limitations; hence, a multifaceted approach is advised to improve udder health. Ultrasound is an economical, rapid tool for detecting mastitis on dairy farms. The PSD and NPVs of mammary were significantly associated with SCS, DIM, SO, season, as well as the scanning plane and udder quarter (*p* < 0.05). The correlation coefficient between pre-milking sagittal PSD and SCC was the highest (*r* = 0.4224, *p* < 0.0001). The pre-milking sagittal PSD of mammary gland value could explain the SCC variation in milk well. Computer-assisted pixel analysis of mammary gland ultrasonograms examination of the udder can aid in diagnosing subclinical mastitis in buffaloes. However, further research is needed to validate the current study's findings and statistical methods with large sample size and data.

## Data Availability Statement

The raw data supporting the conclusions of this article will be made available by the authors, without undue reservation.

## Ethics Statement

The animal study was reviewed and approved by the Ethical Committee of the Hubei Research Center of Experimental Animals. Written informed consent was obtained from the owners for the participation of their animals in this study.

## Author Contributions

LY and XZ: conceptualization. XZ, ZA, KN, and WW: methodology. PN and SG: software and data curation. XZ and MA: investigation. XZ: writing—original draft preparation. MA: writing—review and editing. LY: supervision, project, and funding acquisition. All authors have read and agreed to the published version of the manuscript.

## Funding

China Agriculture Research System of MOF and MAR supported this research work.

## Conflict of Interest

The authors declare that the research was conducted in the absence of any commercial or financial relationships that could be construed as a potential conflict of interest.

## Publisher's Note

All claims expressed in this article are solely those of the authors and do not necessarily represent those of their affiliated organizations, or those of the publisher, the editors and the reviewers. Any product that may be evaluated in this article, or claim that may be made by its manufacturer, is not guaranteed or endorsed by the publisher.
